# Interactive workshops as a learning and teaching method for primary healthcare nurses

**DOI:** 10.4102/hsag.v26i0.1643

**Published:** 2021-12-10

**Authors:** Eva Mukurunge, Marianne Reid, Annali Fichardt, Mariette Nel

**Affiliations:** 1 School of Nursing, Faculty of Health Sciences, University of the Free State, Bloemfontein, South Africa; 2 Department of Biostatistics, Faculty of Health Sciences, University of the Free State, Bloemfontein, South Africa

**Keywords:** interactive workshops, learning and teaching, primary healthcare nurses, role play, group discussions, scenarios

## Abstract

**Background:**

Traditionally, learning by and teaching for primary healthcare (PHC) nurses use didactic, teacher-centred approaches. Hence, the feasibility of interactive workshops in non-threatening PHC environments to refresh nurses’ knowledge on patient care needs exploring.

**Aim:**

To describe interactive workshops as a learning and teaching method for PHC nurses.

**Setting:**

Primary healthcare clinics.

**Methods:**

Systematic literature search followed by an exploratory experimental pre or post-test control group design. Random clinic sampling (*n* = 26) led to clinic inclusion at the control (*n* = 5) and experimental (*n* = 5) sites. Nurses (*n* = 42) were conveniently selected for the control (*n* = 21) and experimental (*n* = 21) groups. Experimental participants (*n* = 21) attended interactive workshops (*n* = 5) where various strategies were applied, whilst addressing key diabetes messages. Both groups completed a questionnaire aligned to diabetes messages pre- and post-workshop. Additionally, a Likert scale questionnaire was posed to the experimental group post-workshop. Data was analysed statistically and presented as descriptive statistics, frequencies and percentages.

**Results:**

Articles reviewed (*n* = 20) identified types of interactive activities, role players, learning content covered, feasibility and duration of the interactive workshops. Pre or post-testing results of the workshops participants indicate improved knowledge related to peripheral sensation (0.03) and (< 0.01). Results from the questionnaire revealed participants’ satisfaction with the interactive workshops.

**Conclusion:**

Interactive workshops as a learning and teaching method could lead to change in knowledge, and participant satisfaction. However, using a combination of interactive workshops and other teaching modalities may enhance learning and teaching further.

**Contribution:**

Interactive workshops are a feasible instructional method during refresher courses for healthcare providers.

## Introduction

Nurses cannot function effectively in an ever-evolving health environment unless they continuously update their knowledge. Both informal and formal platforms exist to update knowledge. Informal platforms include mentoring and coaching, with interactive workshops being a formal method to acquire knowledge, skills and attitudes.

Facilitators of workshops have generally been using the didactic method of information delivery. According to Ross, Hauer and Van Melle (2018:2), it is imperative that health education shifts towards a learner-centred approach, which would ensure that learners engage with study material as they work in groups to construct meaning. When interactive approaches are employed, critical thinking skills are cultivated, and could produce, as an outcome, lifelong learners who are competent enough to manage patients in an environment characterised by an ever-evolving disease burden (Rezgui, Mhiri & Ghedira 2017:397). Consequently, it is essential that workshops are designed in a manner that incorporates interactive activities.

Interactive workshops involve a collection of activities that are organised for groups of participants who have to work together to explore and solve problems presented to them collectively. These activities include small-group discussions, scenarios, simulation, videos, games and role plays. Interactive workshops are conducted over a specific period and in a certain location. In the case of nurses, the workshops can be conducted away from real patients, which affords a safe environment that promotes maximum learning, because participants can afford to make mistakes without the fear of harming patients (So et al. 2019:42). The number of participants in an interactive workshop should not exceed 15, to allow every participant to actively engage with the study material (Elliot et al. 2016:73).

In interactive workshops, small-group discussions enhance the building up of trust and confidence, and sharing of ideas amongst participants in a non-judgemental environment (Dorri et al. 2019:5). Scenarios are presented to participants to solve problems and promote critical thinking and, thereby, prolong retention of knowledge. Simulation is a method of imitating real-life scenarios in a guided manner in a controlled environment (Yu & Kang 2017:42). Furthermore, simulation promotes participation without fear of making mistakes that could harm the patient (West 2016:125). Videos enhance learning, because they appeal to the senses of hearing and sight, which gives the participant a clearer perspective on the concept being learnt. Games bring fun to learning and, thus, learning is assumed to take place with less effort (Ballou et al. 2017:S204). Role plays, in turn, encourage experiential learning, which boosts confidence regarding the application of skills (Doherty et al. 2018:523). Such workshops are presented within a specific context.

The healthcare delivery system of South Africa is primary healthcare (PHC) -driven, and nurses are the main healthcare providers (Austin-Evelyn et al. 2017:online). The system is confronted by an ever-increasing burden of non-communicable diseases (NCDs), amongst others, diabetes (Pheiffer et al. 2018:online). As a result, PHC nurses need to be competent in managing patients living with chronic NCDs, such as diabetes.

Interactive workshops can be a viable method for continuing professional development of PHC nurses in relation to diabetes management in a South African context (Reid et al. 2018:122). The study by Reid et al. (2018:128) in the Free State province of South Africa identified key diabetes messages to be conveyed to nurses. The messages focus on diabetes being a controllable disease, the importance of regular meals, exercise, medication adherence and weight loss, and attitude towards being diagnosed with diabetes. However, the feasibility of interactive workshops as a learning and teaching method for PHC nurses had not been determined.

## Methods

A phased research approach structured the present study. The phases consisted of a systematic literature search (phase 1), followed by a pilot of an interactive workshop that was developed (phase 2).

### Phase 1: Systematic literature search

The first three steps of the systematic review process (eds. Higgins & Green 2011:online) were adapted to inform the systematic literature search. The focused review question that was used for the literature search was as follows: *What is the feasibility of interactive workshops for health professionals?* The variables in the question are based on the PICO format, and formulated as Population (P) – health workers; Intervention (I) – interactive workshops; Control (C) – routine lectures; Outcome (O) – feasibility of interactive workshops as evidenced by changes in knowledge.

A subject librarian assisted in a systematic search on 15 electronic databases namely, CINAHL, MEDLINE, Academic Search Complete, PsycINFO, Health Source: Nursing/Academic Edition, ERIC, Africa-Wide Information, CAB Abstracts, SocINDEX, MasterFILE Premier, American Doctoral Dissertations, Business Source Complete, PsycARTICLES, Communication & Mass Media Complete and EconLit. The search was limited to articles published between March 2008 and November 2020.

In order to retrieve abstracts from the databases, search words used in the search string were constructed from the review question. Table 1 depicts the search words used in the search string.

**TABLE 1:** Search words used in the search string.

First set	Second set	Third set
([nurs * or ‘healthcare worker*’ or ‘health care worker*’ or carer* or caregiver* or ‘care giver*’ or ‘healthcare provider*’ or ‘health care provider*’ or ‘healthcare professional*’ or ‘health care professional*’ or doctor* or physician* or ‘general practitioner*’ or ‘family practitioner*’]) not (patient* or ‘nursing home*’ or doctoral*)	(interact* or collaborat* or group*) n3 (workshop* or discus* or dialog* or engag* or outreach* or platform* or train* or educat* or session* or activit* or program* or interven*)	ti ([interact* or collaborat* or group*] n3 [workshop* or discus* or dialog* or engag* or outreach* or platform* or train* or educat* or session* or activit* or program* or interven*] and [nurs* or ‘healthcare worker*’ or ‘health care worker*’ or carer* or caregiver* or ‘care giver*’ or ‘healthcare provider*’ or ‘health care provider*’ or ‘healthcare professional*’ or ‘health care professional*’ or doctor* or physician* or ‘general practitioner*’ or ‘family practitioner*’])

Initially, titles and abstracts of sampled articles were filtered according to specified inclusion criteria. The study population had to consist of healthcare professionals being subjected to interactive workshops as a platform for professional development, as well as that the outcome of the interactive workshops had to indicate change in knowledge. Research briefs and editorials were excluded. Full articles of the abstracts that met the inclusion criteria were accessed, to determine if the articles did indeed comply with all inclusion criteria. Additionally, reference lists of identified articles were screened for further articles. The first author (E.M.) was responsible for the initial filtering of titles and abstracts and full articles, with other authors (M.R. and A.F.) verifying compliance to eligibility criteria.

### Phase 2: Piloted workshop

The interactive workshops were conducted during January 2019. Random sampling of PHC clinics (*n* = 26), in two semi-urban towns in a sub district of the Free State province of South Africa was done. Five PHC clinics were randomly sampled, each for the control and the experimental sites. Conveniently selected professional, enrolled or auxiliary nurses (*n* = 42) were allocated to control (*n* = 21) and experimental (*n* = 21) sites. or The inclusion criteria relevant to nurse participants is presented in Box 1.

Box 1 Inclusion criteria relevant to nurse participants.

Employed as nurse (professional, enrolled or auxiliary) by the Free State Department of Health

Provides care to patients diagnosed with any type of diabetes at the sampled clinics

Willing to participate in the research

Both experimental and control groups completed the same pre-test consisting of an author-developed questionnaire comprising 12 questions that were aligned to the pre-identified key diabetes messages and related literature. This questionnaire did not undergo validity testing.

Each PHC clinic in the experimental group underwent a single, hour long workshop. The content of the programme focused on key diabetes messages (See messages depicted in Table 3). All PHC clinics in the experimental group went through the exact same programme. All the workshops (*n* = 5) were facilitated by the first author (EM), who is an experienced PHC educator. The control group did not receive any interactive workshops.

The post-test comprised the same questions as the pre-test, posed to experimental group participants within 2 h of completing the interactive workshop. This group of participants also assessed nine statements on a Likert scale, which provided them with an opportunity to evaluate the workshop. Control group participants also completed the post-test within 2 h after the completion of the pre-test.

### Ethical considerations

Ethical approval was obtained from the University of the Free State, reference number: UFS-HSD2017/0640, and permission was obtained from relevant role players at the Free State Department of Health. The three broad ethical principles of the Belmont Report (The National Commission for the Protection of Human Subjects of Biomedical and Behavioral Research 1979) were upheld in this study. These include: the principle of beneficence, principle of respect for persons, and the principle of justice (Speziale & Carpenter 2007:61). In order to ensure beneficence, the interactive diabetes workshops were facilitated at the PHC clinics in safe and familiar environments for the participants to ensure security and protection of the participants from harm and discomfort. Full information about the study was given to all participants, as an indication of respect for persons, who were free to make an informed decision on whether or not to participate. The principle of justice was upheld by ensuring privacy in all the data gathered from participants. The participants used codes instead of their actual names. Data collected were kept in a locked cupboard and files on the computer were password protected. There was no discrimination during the selection process of participants for the study because those that were available and were willing to participate were selected.

## Results

Results are presented according to the phased approach followed in the study. Data from the literature search will be presented first, followed by data relating to the piloted interactive workshop.

### Phase 1: Systematic literature search

**FIGURE 1:**
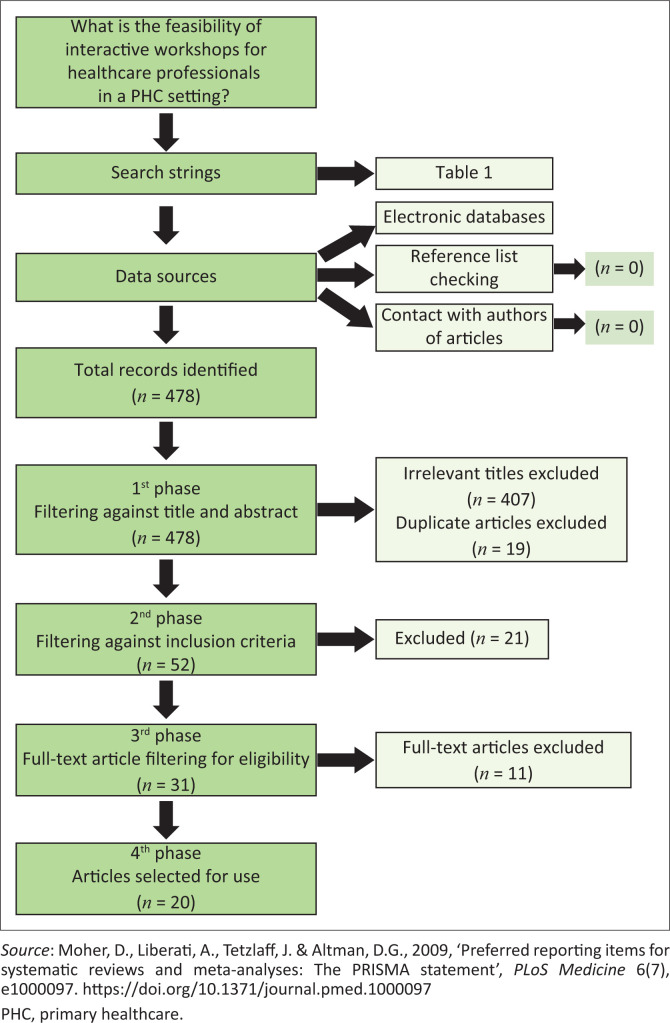
PRISMA flow chart showing the systematic literature search.

Figure 1 depicts the flow diagram of the systematic literature search. From the records identified initially (*n* = 478), a large number (*n* = 407) were excluded because of them failing to meet the inclusion criteria. A comprehensive screening of full-text articles (*n* = 31), led to only 20 articles meeting all the inclusion criteria. Contents of these 20 articles were then categorised to identify the type of interactive activities, role players, learning content covered, feasibility and duration of interactive workshops (Online Appendix 1).

A summary of the 20 articles that were selected is available in Online Appendix 1. *Interactive activities* extracted from the selected articles and usable in the context of this study include small-group discussions, role play, case studies, simulation, demonstration and problem-solving activities, such as Kagan’s rally coach, timed pair share, and rally robin. The *duration* of the interactive workshops reported in extracted articles show a great variance, from an hour to longer interactive activities spread over up to 8 weeks. The duration proposed for the interactive workshops of this study was an hour, because of local circumstances at PHC clinics. Each selected study had its own *learning content* focus, whilst this study’s content focused on the six key diabetes messages established by Reid et al. (2018:128) as part of previous research conducted in the Free State province of South Africa amongst patients diagnosed with diabetes, community members, and healthcare workers caring for these patients (see Box 2).

Box 2 Key diabetes messages.

Diabetics should:

- Eat small, regular meals
- Aim to walk fast at least 30 min most days
- Lose weight as prescribed.
- Take medication as prescribed

Diabetes can be controlled and complications can be prevented

Diabetics can enjoy a normal life

*Source*: Reid, M., Walsh, V., Raubenheimer, J., Bradshaw, T., Pienaar, M., Hassan, C. et al., 2018, ‘Development of a health dialogue model for patients with diabetes: A complex intervention in a low/middle-income country’, *International Journal of African Nursing Sciences* 8(1), 122–131. https://doi.org/10.1016/j.ijans.2018.05.002

### Phase 2: Piloted interactive workshops

Table 2 indicates that the demographic profiles of participants in the experimental and control groups were to a large extent similar. The PHC nurses who were selected to participate in both groups were practising in public PHC clinics, and were exposed to similar working conditions. The majority were mature women who had obtained diplomas as their highest academic qualification. Registered nurses form the backbone of the sampled PHC clinics.

**TABLE 2:** Demographic profile of participants (*n* = 42).

Variable	Experimental group (*n* = 21)			Control group (*n* = 21)	
	*n*	%		*n*	%
**Gender**					
Female	18	85.7		18	85.7
Male	3	14.3		3	14.3
**Age**					
20–50	4	19.0		5	23.0
51–70	17	80.9		16	76.2
**Highest academic qualification**					
Certificate	5	23.0		8	38.1
Diploma	11	52.4		10	47.6
Bachelor’s degree	5	23.8		3	14.0
**Employment position**					
Registered nurse	14	66.7		11	52.4
Enrolled nurse	4	19.0		2	9.5
Auxiliary nurse	3	14.3		8	38.1

Interactive activities within this workshop included case scenarios, role plays, small group discussions, Kagan’s round robin, rally robin, rally coach, and timed pair share. The workshop programme may be requested from the authors.

Results of pre- and post-workshop testing of key diabetes messages are depicted in Table 3. These pre or post- test questions were presented as statements.

**TABLE 3:** Participant responses to key diabetes messages in control (*n* = 21) and experimental (*n* = 21) groups with related *p* values.

Question (Key diabetes message)		Control						Experimental						Changes from pre- to post-test		
		Pre-test phase			Post-test phase			Pre-test phase			Post-test phase			Statistical test used		
		True	False	Unsure	True	False	Unsure	True	False	Unsure	True	False	Unsure	McNemar		Fisher’s exact test/Chi-square
														*p* value for control group	*p* value for experimen-tal group	*p* values across control and experimental groups
1. In order to avoid foot ulcers, people with diabetes should wear shoes with pointed tips. (Diabetes can be controlled and complications can be prevented.)	*n*	1.0	**20.0**	0.0	2.0	**18.0**	1.0	1.0	**20.0**	0.0	2.0	**19.0**	0.0	0.59	0.32	1.000
	%	4.8	**95.2**	-	9.5	**85.7**	4.8	4.8	**95.2**	-	9.5	**90.5**	-	-	-	-
2. People with diabetes need regular eye check-ups to prevent or delay onset of diabetic retinopathy. (Diabetes can be controlled and complications prevented.)	*n*	**19.0**	2.0	0.0	**20.0**	1.0	0.0	20.0	0.0	1.0	**21.0**	0.0	0.0	0.56	0.47	0.490
	%	**90.5**	9.5	-	**95.2**	4.8	-	95.2	-	4.8	**100.0**	-	-	-	-	-
3. Brisk walking daily for 30 min is good for lowering blood glucose levels. (Diabetics should aim to walk fast for at least 30 min most days.)	*n*	**17.0**	1.0	3.0	**17.0**	1.0	3.0	**20.0**	0.0	1.0	**21.0**	0.0	0.0	1.00	0.47	0.230
	%	**80.9**	4.8	14.3	**80.9**	4.8	14.3	**95.2**	-	4.8	**100.0**	-	-	-	-	-
4. Exercise increases glucose uptake by the muscles, and insulin sensitivity. (Diabetics should aim to walk fast for at least 30 min most days.)	*n*	**7.0**	6.0	8.0	**12.0**	3.0	6.0	**12.0**	5.0	4.0	**16.0**	5.0	0.0	0.17	0.18	1.000
	%	**33.3**	28.6	38.1	**57.1**	14.3	28.6	**57.1**	23.8	19.1	**76.2**	23.8	-	-	-	-
5. Weight loss in obese diabetes patients can improve blood glucose levels. (Diabetics should lose weight as prescribed.)	*n*	**20.0**	1.0	0.0	**18.0**	3.0	0	**15.0**	4.0	2.0	**20.0**	1.0	0.0	0.32	0.21	1.000
	%	**95.2**	4.8	-	**85.7**	14.3	-	**71.4**	19.1	9.5	**95.2**	4.8	-	-	-	-
6. Poor control of blood glucose can lead to loss of sensation in the periphery. (Diabetes can be controlled and complications can be prevented.)	*n*	**17.0**	3.0	1.0	**18.0**	2.0	1.0	**13.0**	6.0	2.0	**20.0**	0.0	1.0	0.95	0.03٭	1.000
	%	**80.9**	14.3	4.8	**85.7**	9.5	4.8	**61.9**	28.6	9.5	**95.2**	-	4.8	-	-	-
7. Meals for people with diabetes should be small and frequent. (Diabetics should eat small, regular meals.)	*n*	**21.0**	0.0	0.0	**18.0**	1.0	2.0	**20.0**	1.0	0.0	**21.0**	0.0	0.0	0.38	0.47	1.000
	%	**100.0**	-	-	**85.7**	4.8	9.5	**95.2**	4.8	-	**100.0**	-	-	-	-	-
8. People with diabetes should eat unrefined carbo-hydrates, like whole wheat grains, vege-tables and fruits. (Diabetics should eat small, regular meals.)	*n*	**21.0**	0.0	0.0	**18.0**	1.0	2.0	**20.0**	1.0	0.0	**20.0**	1.0	0.0	0.38	1.00	1.000
	%	**100.0**	-	-	**85.7**	4.8	9.5	**95.2**	4.8	-	**95.2**	4.8	-	-	-	-
9. Insulin is the first line drug of choice for Type 2 diabetes. (Medications must be taken as prescribed.)	*n*	8.0	**10.0**	3.0	5.0	**12.0**	4.0	17.0	**3.0**	1.0	5.0	**12.0**	4.0	0.26	0.07	0.040*
	%	38.1	**47.6**	14.3	23.8	**57.1**	19.1	80.9	**14.3**	4.8	23.8	**57.1**	19.1	-	-	-
10. Insulin administered in an overused site will be absorbed faster. (Medications must be taken as prescribed.)	*n*	4.0	**13.0**	4.0	5.0	**10.0**	6.0	5.0	**9.0**	7.0	10.0	**9.0**	2.0	0.80	0.17	1.000
	%	19.1	**61.9**	19.1	23.8	**47.6**	28.6	23.8	**42.9**	33.3	47.6	**42.9**	9.5	-	-	-
11. Depression is common in patients with diabetes. (Diabetics can enjoy a normal life.)	*n*	**16.0**	3.0	2.0	**15.0**	5.0	1.0	**8.0**	8.0	5.0	**20.0**	0.0	1.0	0.39	< 0.01*	0.001*
	%	**76.2**	14.3	9.5	**71.4**	23.8	4.8	**38.1**	38.1	23.8	**95.2**	-	4.8	-	-	-
12. With much support from family and healthcare professionals, people with diabetes can cope with the demands of diabetes. (Diabetics can enjoy a normal life.)	*n*	**21.0**	0.0	0.0	**19.0**	1.0	1.0	**20.0**	1.0	0.0	**21.0**	0.0	0.0	0.59	0.82	1.000
	%	**100.0**	-	-	**90.5**	4.8	4.8	**95.2**	4.8	-	**100.0**	-	-	-	-	-

* , Statistical significance ≤ 0.05.

Statistical analysis of data was done to produce descriptive statistics, frequencies and percentages. The changes from pre- to post-tests were calculated and described by means of McNemar’s test for paired data.

Statistically significant results from pre- to post-testing relate to key diabetes messages – the experimental group indicated improved knowledge related to peripheral sensation (0.03) and depression (< 0.01).

The five-point Likert scale which was used to evaluate the workshop revealed that the participants in our study expressed satisfaction with the interactive workshops. They perceived that their knowledge of diabetes management had been refreshed in a relaxed, non-threatening environment.

### Rigour

The criteria used to ensure rigour in the phases of the study included truth value, consistency, neutrality and applicability. A clearly structured review question confirmed truth value. An audit trial strengthened consistency, and stipulated inclusion criteria supported neutrality throughout the phases. The systematic presentation assists readers to decide about the applicability of findings to other contexts.

## Discussion

This study investigated interactive workshops as a learning and teaching method for PHC nurses. However, without a sound theoretical underpinning, the value of these workshops in a nursing environment cannot be interpreted. In order for interactive workshops to be effective, they have to be underpinned by four theories: constructivism, constructive alignment, scaffolding, and authenticity (Biggs 2001:225; Bruner 1975:4; Roach, Tilley & Mitchell 2018:497; Vygotsky 1978:3).

*Constructivism* is a theory that posits that learning takes place whilst participants work in groups in their cultural contexts to construct meaning of a new concept (Vygotsky 1978:3). In the present study, PHC nurses were challenged to construct meaning in relation to concepts they had been exposed to previously, although still in a familiar context. Constructivism acknowledges participants’ prior knowledge, on which new information is built. Therefore, as the participants construct new knowledge, they reflect on the learnt material and make connections to their prior information, until meaning is made and ownership of the newly constructed knowledge is established (Bada 2015:66). This principle was integrated during the workshops that were presented, with nurses’ understanding of key diabetes messages being used as a platform for new information. According to constructivism, learning is a social activity, which implies the facilitator has to ensure that the culture referenced and the language used during the workshop are common to the participants. The PHC context in which workshops were held naturally lent itself to culture and language sensitivity.

Participants enter interactive workshops with already existing knowledge. According to the theory of *scaffolding,* the facilitator has to take participants from their level of independent performance to a higher level of dependency, where, through maximal support, new levels of independence are achieved (Bruner 1975:4). The higher level of dependency is what Vygotsky refers to as the zone of proximal development; it is in the zone of proximal development that the facilitator has to offer support. In the case of interactive workshops, participants could be offered reading material, coaching and demonstrations to foster understanding of new concepts and skills (Vygotsky 1978:7). During the relative short contact time, the facilitator was able to clarify the understanding of key diabetes messages with nurses.

The activities that participants engage in the zone of proximal development enhance their critical reasoning skills for problem-solving. Once they manage to solve a problem, their motivation and confidence to address tasks that are more challenging are boosted. However, as learning takes place, the support that the participants receive from the facilitator is tapered off, until they perform independently. All the interactive activities and the support have to occur in an authentic learning environment (Vygotsky 1978:7).

*Authenticity* refers to the requirement that the activities and the learning environment approximate the real environments closely (Roach et al. 2018:497), as was the case during diabetes-related group discussions in the reported interactive workshops. The skills and knowledge are acquired in a manner similar to their application in the real environment. In order for interactive workshops to be effective and learning to be enhanced, activities employed should cater for participants’ different learning styles. The different learning styles that should be considered are visual, auditory, reading and kinaesthetic styles. Using activities that cater for different learning styles enhances participation, engagement and learning (Quinn et al. 2018:358). The reported interactive workshops, therefore, also made use of a variety of activities, such as case studies, music and drawing.

The interactive activities included by this study in a PHC setting have also been used in various other settings where nurses provide care, which seems to indicate the feasibility of using such activities during workshops. In Turkey (Tiryaki & Cinar 2016:163), nurses’ knowledge levels of neonatal intensive care improved after they had been trained through interactive workshops. A systematic review conducted by Rønning and Bjørkly (2019:415) suggested using role play to enhance nurses’ therapeutic and communication skills. Role play and subsequent group discussions also proved to enhance interview skills of psychiatric nurses in Taiwan (Chen 2016:146), whereas a study conducted in Brazil (Cogo et al. 2016:1163) found that, when case studies and role plays were used, nursing students were encouraged to actively search for learning, whilst experiencing a closer link between theory and practice. However, interactive activities do not always lead to superior learning outcomes, specifically when used in isolation, rather than in combination with traditional lecturing (Heidarzadeh, Heidarzadeh & Azadi 2020:940) or other teaching modalities.

### Limitations of the study

The small sample size does not allow for generalisation of results to a wider nurse population. Because of service delivery challenges, a single workshop with short pre- to post-test interval time could influence the reliability of results.

## Conclusion

This research suggests that interactive workshops could be a feasible learning and teaching method for PHC nurses. A systematic literature search identified small-group discussions, role play, case studies, simulation, demonstration and problem-solving activities, such as Kagan’s rally coach, timed pair share and rally robin, as the most suitable interactive workshop activities. Taking the current situation of the coronavirus disease 2019 (COVID-19) pandemic into consideration, contemporary virtual interactive platforms such as Microsoft teams, Zoom or WhatsApp can be explored to present interactive workshops. The activities used within the reported workshops, which included case scenarios, role plays, small group discussions, Kagan’s round robin, rally robin, rally coach and timed pair share, resulted in improved knowledge after the workshop, and satisfaction with the workshops by the participants. However, a healthcare setting determines which role players could be involved in interactive workshops, the specific activities used, the duration of workshops, as well as the learning content and platform. Using interactive workshops and other modalities as learning and teaching methods should be embraced, because they enhance knowledge refreshment, communication skills, critical-thinking skills, and decision-making skills.
